# Neutrophil-targeted nanoparticles delivering sivelestat alleviate cerebral ischemia-reperfusion injury by suppressing NETosis

**DOI:** 10.1016/j.mtbio.2026.103408

**Published:** 2026-06-29

**Authors:** Shuyu Wu, Xiaoli Sun, Xiai Luo, Yang Li, Jinlong Wan, Chuanwu Xiong, Qingmin Chen, Xiaohong Lv, Ruwei Jie, Sainawar Tursun, Jianjun Ma, Qingchun Mu, Longguang Tang, Hongzhi Gao

**Affiliations:** a Department of Neurosurgery, The Second Affiliated Hospital of Fujian Medical University, Quanzhou, 362000, China; b Hunan Province Key Laboratory for Synthetic Biology of Traditional Chinese Medicine, School of Pharmaceutical Sciences, Hunan University of Medicine, Huaihua, 418000, China; c Department of Pharmacy, Center for Regenerative and Aging Medicine, The Fourth Affiliated Hospital of School of Medicine and International School of Medicine, International Institutes of Medicine, Zhejiang University, Yiwu, 322000, China; d Department of Neurosurgery, Hainan General Hospital, Haikou, Hainan, 570000, China; e Affiliated Gaozhou People's Hospital, Guangdong Medical University, Maoming, 525200, China; f Department of Orthopaedic Surgery, Sir Run Shaw Hospital, Zhejiang University School of Medicine, Zhejiang Key Laboratory of Mechanism Research and Precision Repair of Orthopaedic Trauma and Aging Diseases, Hangzhou, Zhejiang, 310000, China; g Department of Orthopaedic Surgery, The Fourth Affiliated Hospital of School of Medicine, and International School of Medicine, International Institutes of Medicine, Zhejiang University, Yiwu, 322000, China; h Shanxi Province Cancer Hospital, Shanxi Hospital Affiliated to Cancer Hospital, Chinese Academy of Medical Sciences, Cancer Hospital Affiliated to Shanxi Medical University, Taiyuan, 030000, China

## Abstract

As a leading cause of disability and mortality, ischemic stroke (IS) constitutes 80–90% of all stroke cases, underscoring the need for effective therapies. The pathogenesis of IS critically involves neutrophil extracellular traps (NETs), with neutrophil elastase (NE) serving as a core enzymatic driver of NETosis and a prime therapeutic target. Herein, we report a neutrophil-targeted nanotherapeutic strategy designed to exploit the pharmacological action of the NE inhibitor Sivelestat (SIV). Our engineered nanoparticles (T-SIV) feature a formyl peptide receptor (FPR)-specific ligand for neutrophil targeting and reactive oxygen species (ROS)-responsive thioketal linkages for site-specific drug release. Evaluation in MCAO mice and OGD/R-injured PC12 cells confirmed the efficacy of T-SIV, which conferred multimodal neuroprotection by reducing cerebral infarct volume, alleviating edema, and restoring neurological function. The therapeutic mechanism entails the suppression of NE-dependent NETosis coupled with a shift in microglial polarization toward the anti-inflammatory M2 phenotype, resulting in dual modulation of neuroinflammation. This study thus introduces a neutrophil-hitchhiking nanoplatform that synergistically disrupts the NE–NETs axis and reprograms the inflammatory microenvironment, presenting a transformative approach for the treatment of ischemic stroke.

## Graphical abstract

**Figure:**
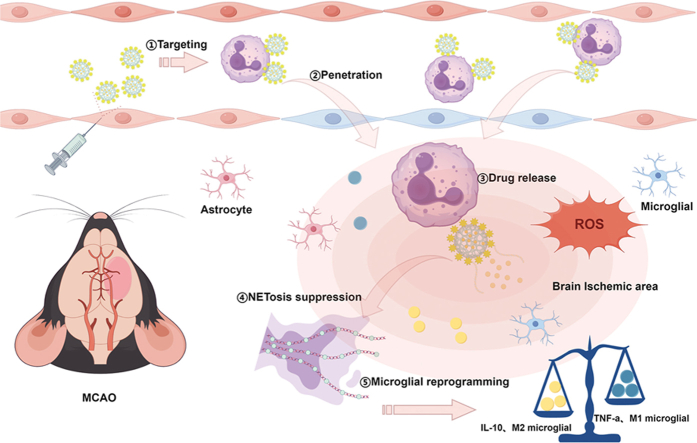

## 1 Introduction

Stroke, the second leading cause of global mortality, not only constitutes a primary etiology of disability and death in working-age populations but also accounts for approximately 6 million annual deaths worldwide [1]. In China, the disease burden exceeds 13 million stroke survivors, imposing substantial economic strain on both affected families and societal healthcare systems [2]. Ischemic stroke (IS), representing 80%-90% of all stroke cases, manifests its pathological hallmark as thromboembolism-induced cerebral blood flow interruption [3]. Current therapeutic strategies predominantly involve intravenous thrombolysis (administered within a strict 4.5- to 6-h window) combined with endovascular mechanical thrombectomy, both targeting revascularization of ischemic territories [4]. Nevertheless, the constrained treatment eligibility (only 5-10% of patients meeting thrombolytic criteria) coupled with a 12-27% incidence of hemorrhagic transformation post-recanalization substantially compromises therapeutic efficacy in IS management [5].

During the IS pathological cascade, cerebral ischemia-reperfusion injury (CIRI) emerges as a pivotal secondary injury mechanism influencing clinical outcomes. Neutrophils, as the first immune cells infiltrating ischemic regions, participate in CIRI regulation through neutrophil extracellular traps (NETs) formation [6]. These three-dimensional reticular structures, composed of histone-decorated dsDNA scaffolds and numerous granular proteins, exert dual biological effects: while serving innate immune defense functions, they exacerbate blood-brain barrier disruption by amplifying inflammatory cascades [7,8]. Notably, neutrophil elastase (NE) functions not merely as a structural component of NETs but also acts as a crucial effector molecule driving excessive reactive oxygen species (ROS) generation [9]. Experimental evidence demonstrates that pharmacological inhibition of NE activity significantly reduces cellular apoptosis in ischemic regions, whereas enzymatic degradation of NETs by DNase I markedly attenuates cerebral infarct volume [10,11]. These collective findings underscore the therapeutic potential of targeting the NE-NETs axis in IS management [6].In line with this concept, recent advances have further validated NETosis as a promising therapeutic target. He et al. [12]systematically elucidated the triple detrimental mechanisms of NETs in ischemic stroke, while Yin et al. [13]and Tang et al. [14] developed innovative nanoplatforms that inhibit NETosis and promote neurovascular remodeling.

Sivelestat is a potent and selective small-molecule inhibitor of NE. It acts by competitively occupying the enzyme's catalytic site, thereby producing anti-inflammatory effects, which have been validated in the clinic for systemic inflammatory conditions [[15], [16], [17]]. Although the drug was approved by the China National Medical Products Administration (NMPA) in 2020 for respiratory complications [16], Sivelestat suffers from a short half-life and lacks cell-specific targeting capability. Furthermore, the presence of the blood–brain barrier (BBB) severely restricts the drug concentration reaching the cerebral ischemic tissue. Sustained administration is required to maintain effective concentrations, which in turn increases systemic toxicity and the risk of adverse events. Collectively, these factors substantially compromise the delivery efficiency of this small-molecule agent for the treatment of ischemic stroke [18]. Conventional dose escalation cannot overcome these limitations without incurring unacceptable toxicity, highlighting the urgent need for a targeted delivery strategy. Experimental evidence confirms that the free-form Sivelestat fails to achieve satisfactory therapeutic outcomes in murine cerebral ischemia models [19].

The advent of nano-targeted drug delivery systems provides innovative strategies to overcome these delivery challenges. As early responders, neutrophils infiltrate lesion sites within 4-6 h post-inflammatory initiation and peak at 2-3 days [20]. Administering therapeutics targeting inflammation-homing cells during this early window leverages neutrophil migration for precise lesion delivery—a strategy termed “cellular hitchhiking” [21]. Nanoencapsulation of small-molecule drugs with targeted ligand modification enhances nanoparticle interactions with circulatory immune cells, thereby exploiting neutrophil inflammatory chemotaxis for site-specific accumulation. Polymeric nanoparticles (PNPs), as core nanocarriers, achieve stable drug loading in particle cores or surface matrices through covalent conjugation [22], while enabling targeted drug accumulation at lesions via ligand-receptor interactions [23]. Studies confirm PNPs not only significantly enhance payload aqueous solubility and chemical stability but their exceptional biocompatibility and facile synthesis establish them as ideal delivery platforms for ischemic stroke research [22,23].

**Fig. 1:**
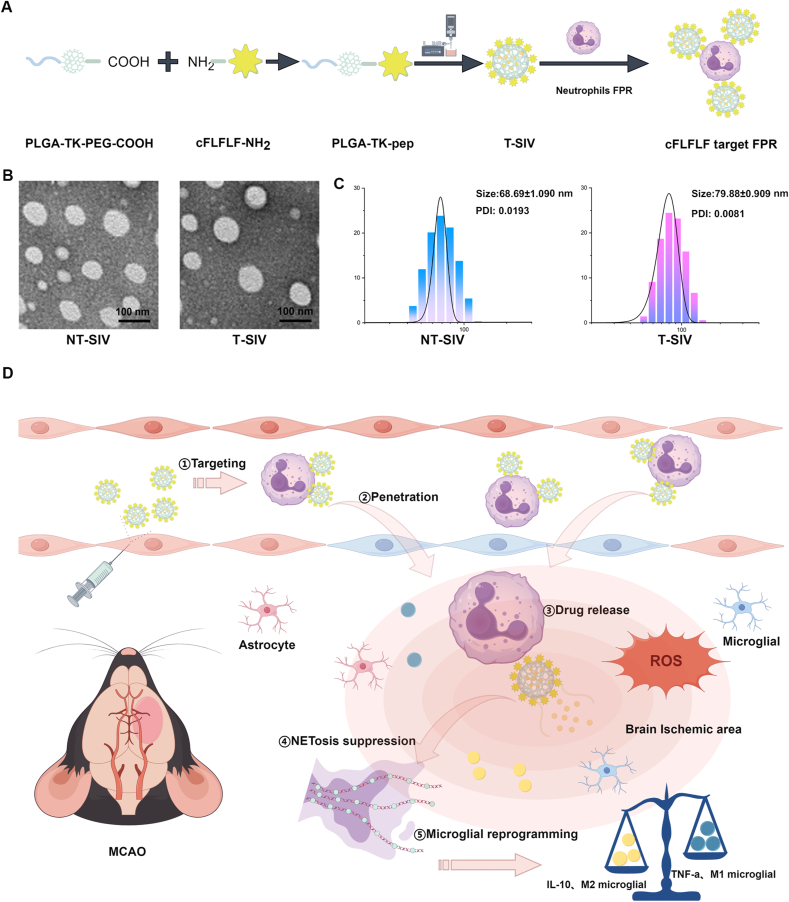
**Fabrication, characterization, and mechanism of T-SIV nanoparticles.** (A) Schematic of neutrophil-targeted nanoparticles loaded with Sivelestat (T-SIV). (B) TEM micrographs demonstrating monodisperse spherical morphology of T-SIV and NT-SIV with mean diameters approximating 100 nm. (C) Hydrodynamic diameters measured by DLS: 79.9 ± 0.91 nm (T-SIV) vs. 68.7 ± 1.09 nm (NT-SIV). (D)Mechanistic cascade of T-SIV:① **Neutrophil targeting:** Post-intravenous administration via FPR-mediated neutrophil targeting; ② **BBB penetration:** Neutrophil-driven transport across the blood-brain barrier with ischemic region enrichment; ③ **ROS-triggered drug release:** Thioketal bond cleavage in high-ROS microenvironment enables controlled SIV liberation; ④ **NETosis suppression:** SIV-mediated neutrophil elastase inhibition attenuates NET formation, concomitant with pro-inflammatory cytokine (e.g., TNF-α) downregulation and anti-inflammatory mediator (e.g., IL-10) upregulation; ⑤ **Microglial reprogramming:** Polarization of microglia from pro-inflammatory M1 phenotype to neuroprotective M2 subtype, ultimately restoring neurovascular unit homeostasis.

Accordingly, this study engineered a neutrophil-targeting nanovector based on poly(lactic-co-glycolic acid) (PLGA) and polyethylene glycol (PEG) copolymers. Surface functionalization with CFLFLF (cinnamyl-F-(D)L-F-(D)LF)—a specific ligand for formyl peptide receptors (FPRs) on neutrophils [24]-enables precise targeting. Incorporation of ROS-responsive thioketal linkers ensures triggered sivelestat release in ischemic regions with elevated ROS concentrations. Using middle cerebral artery occlusion (MCAO) models, we systematically evaluated neutrophil-targeting efficacy and assessed therapeutic outcomes for cerebral ischemia-reperfusion injury through comprehensive neurological scoring and histopathological analyses (Fig. 1D).

## 2 Experimental section

**Materials:** Sivelestat (CAS: 127373-66-4) was procured from Shanghai Bide Pharmaceutical Co., Ltd. PLGA-TK-PEG-COOH (poly(lactic-co-glycolic acid)-thioketal-polyethylene glycol-carboxyl, PEG MW: 2000, PLGA MW: 5000, lactide/glycolide ratio 75:25) was purchased from Xi'an Ruixi Biological Technology Co., Ltd. (Xi'an, China).The FPR-targeting peptide CFLFLF (sequence: cinnamoyl-F-(D)L-F-(D)L-F, purity ≥95%) was custom-synthesized by GenScript (Nanjing, China) under the order number C493GHG130-1. The TUNEL assay kit (Cat. No. 11684817910) was obtained from Roche Diagnostics GmbH. IL-10 (Cat. No. EK0412) and TNF-α (Cat. No. EK0394) ELISA kits were supplied by Shanghai Jingkang Bioengineering Co., Ltd. All aqueous solutions were prepared using Milli-Q ultrapure water. Additional chemical reagents were purchased from Sigma-Aldrich, Thermo Fisher Scientific, or Alfa Aesar.

**Preparation of T-SIV and T-SIV-IR780:** Sivelestat (SIV, 4 mg) and PLGA-Thioketal (TK)-Polyethylene Glycol 2000 (PEG2K)-COOH (40 mg) were dissolved in tetrahydrofuran (THF, 2 mL). Under magnetic stirring (800 rpm), ultrapure water (2 mL) was introduced via dropwise addition. After 12-h THF evaporation in a fume hood, EDC (10 mg) in MES buffer (2 mL, 10 mM, pH 6.0) was added to activate carboxyl groups for 30 min. Subsequently, the FPR-targeting peptide CFLFLF (cinnamyl-F-(D)L-F-(D)L-F, 15 mg) was introduced, followed by continuous stirring for 3 h. The resultant solution underwent triple purification using ultrafiltration centrifuge tubes (MWCO: 3 kDa) at 8000 × g for 15 min, yielding T-SIV nanoparticles (NPs), which were aliquoted and stored at 4°C. For fluorescently labeled T-SIV-IR780 NPs, IR780 aqueous solution (2 mL, 1 mg mL^−1^) was incorporated into the THF phase, following identical subsequent procedures.

**Preparation of NT-SIV and NT-SIV-IR780:** Sivelestat (SIV, 4 mg) and PLGA-TK-mPEG2K (40 mg) were processed following the aforementioned T-SIV synthesis protocol, omitting the EDC-mediated ligand conjugation step. The mixture was directly subjected to ultrafiltration purification (MWCO: 3 kDa, 8000 × g, 15 min) to obtain non-targeted nanoparticles (NT-SIV NPs). For NT-SIV-IR780 NPs, IR780 aqueous solution (1 mg mL^−1^) was co-introduced into the THF phase during nanoparticle formation, maintaining identical parameters to those of T-SIV-IR780 NPs.

**Cellular Uptake Assay:** *(Neutrophil Isolation and Culture):* Bone marrow cells were harvested from murine femurs and tibiae. Following erythrocyte lysis with ACK buffer, neutrophils were isolated via density gradient centrifugation (Histopaque 1077). Cells were seeded at 1 × 10^5^ cells/well in 12-well plates containing coverslips, cultured in RPMI 1640 medium supplemented with 10% FBS for 2 h (37°C, 5% CO_2_) to promote adherence. *(Nanoparticle Treatment):* Cy5-labeled targeted (T-SIV-Cy5) or non-targeted (NT-SIV-Cy5) nanoparticles were added to each well (final concentration: 2 μg/mL), followed by 4-h incubation. *(Immunofluorescence Staining):* Cells were fixed with 4% paraformaldehyde (15 min), permeabilized with 0.1% Triton X-100 (10 min), and incubated with Ly6G primary antibody (1:200, Clone 1A8, BioLegend) at 4°C overnight. Alexa Fluor 488-conjugated secondary antibody (1:500) and DAPI (1 μg/mL) were applied for counterstaining. Fluorescent images were acquired using a confocal laser scanning microscope (CLSM, ZEISS LSM 880) and quantified via ImageJ v1.53. *(Flow Cytometric Analysis):* Harvested cells were stained with CD11b-APC (Clone M1/70) and Ly6G-PE (Clone 1A8) antibodies under light-protected conditions (30 min, 4°C). Cy5+ cell populations were analyzed using a BD FACSCalibur flow cytometer (488 nm laser excitation with 670 nm bandpass filter), with data processed in FlowJo v10. Triplicate independent experiments were performed per group (n = 3).

**Transwell Migration Assay:** *(Blood-Brain Barrier (BBB) Model Establishment):* bEnd.3 cells were seeded in Transwell inserts (polycarbonate membranes, 8 μm pore size, 6.5 mm diameter; Corning Inc.) at 1 × 10^5^ cells/well. Cells were cultured in endothelial cell medium (ECM, 10% FBS) for 48 h. Transepithelial electrical resistance (TEER) was monitored daily using an EVOM2 volt-ohm meter (World Precision Instruments), with BBB integrity defined at TEER ≥300 Ω cm^2^ *(Neutrophil Migration Detection):* Bone marrow-derived neutrophils (1 × 10^5^ cells) and Cy5-labeled nanoparticles (T-SIV-Cy5/NT-SIV-Cy5, 2 μg/mL) were introduced into the upper chamber, while the lower chamber contained fMLP chemoattractant (100 nM, Sigma-Aldrich). Following 4-h incubation (37°C, 5% CO_2_), the upper chamber medium was aspirated. Migrated cells in the lower chamber were immunostained with CD11b-FITC (Clone M1/70, BioLegend) and Ly6G-PE (Clone 1A8, BioLegend), followed by flow cytometric quantification of Cy5+ populations using a BD FACSCalibur system (488/640 nm lasers).

**NIR-II (Second Near-Infrared Window**) **Imaging:** IR780-labeled nanoparticles (T-SIV-IR780/NT-SIV-IR780, 3 mg/kg) were administered via caudal vein injection. Cerebral fluorescence signals were acquired at 1, 4, 8, and 24 h post-injection using a small-animal in *vivo* imaging system (808 nm excitation, 1100 nm emission, 50 ms exposure).

**Immunofluorescence Staining:** Following transcardial perfusion with 4% paraformaldehyde, brain tissues were paraffin-embedded and coronally sectioned (5 μm thickness). Sections were incubated with primary antibodies (Ly6G 1:200, Iba-1 1:500, GFAP 1:1000; Servicebio Biotechnology Co., Ltd.) at 4°C overnight, followed by corresponding secondary antibodies (1:500) and DAPI counterstaining. Fluorescent images were acquired using a confocal laser scanning microscope.

**Middle Cerebral Artery Occlusion (MCAO) Model:** All animal procedures were approved by the Institutional Animal Ethics Committee of Zhejiang University (Approval No.: ZJU20230221) and complied with ARRIVE guidelines. Specific pathogen-free (SPF) male C57BL/6 mice (6-8 weeks old, 20-25 g) were obtained from the university's Experimental Animal Center. Mice were acclimatized for one week under controlled temperature (22 ± 1°C) with 12-h light/dark cycles, provided ad libitum access to food and water. MCAO was induced using an improved Longa filament method [25]:Under 2% isoflurane anesthesia, mice were positioned supine for midline cervical incision (2 cm). The right common carotid artery (CCA), external carotid artery (ECA), and internal carotid artery (ICA) were bluntly dissected. A 6-0 silicone-coated nylon monofilament (diameter: 0.22 mm) was introduced through an ECA arteriotomy and advanced 18 ± 0.5 mm from the CCA bifurcation to occlude the middle cerebral artery origin. Cerebral ischemia was maintained for 90 min followed by filament withdrawal for reperfusion. Incisions were closed with bilayer sutures, with postoperative rewarming at 37°C until consciousness recovery. Sham-operated controls underwent vascular exposure without filament insertion. Eligible mice were randomized into five groups (n = 5/group): **Sham:** Sham surgery + saline (caudal vein injection); **MCAO:** Model + saline; **f-SIV:** Model + free Sivelestat (3 mg/kg, formulated in DMSO/PBS); **NT-SIV:** Model + non-targeted NPs (3 mg/kg); **T-SIV:** Model + targeted NPs (3 mg/kg); Postoperative treatments were administered daily for three consecutive days, with continuous monitoring of body weight and survival status.

**Magnetic Resonance Imaging** (**MRI) Imaging:** At 72 h post-surgery, mice were subjected to 3.0T MRI scanning (Siemens Trio, Siemens Medical Solutions, Germany) with T2-weighted sequences. Hyperintense lesion volume was quantified using ImageJ software through three-dimensional reconstruction of signal abnormalities, with triplicate measurements per group (n = 3).

**Rotarod Test:** Three-day acclimation training was conducted preoperatively with rotational speed progressively increasing from 4 to 20 rpm over a 5-min period. Postoperative assessments were performed daily by measuring the latency to fall from an accelerating rod (4-20 rpm/5 min), with three consecutive trials recorded and averaged for statistical analysis [26].

**Modified Neurological Severity Score (mNSS) Test:** The modified neurological severity score (mNSS, 0-14 scale) was employed to comprehensively assess motor, sensory, and reflex functions, with daily evaluations conducted until postoperative day 14 [27]。

**2,3,5-Triphenyltetrazolium Chloride (TTC) Staining:** At 72 h post-surgery, cerebral tissues were harvested and coronally sectioned (2 mm thickness). The slices were incubated with 2% TTC solution (Leagene Biotechnology Co., Ltd., China) at 37°C under light-protected conditions for 20 min. Infarct volume percentage was quantified using ImageJ software by calculating the unstained area-to-ipsilateral hemisphere ratio (unstained area/ipsilateral hemisphere × 100%), with triplicate measurements per group (n = 3).

**Evans Blue (EB) Permeability Assay:** A 2% Evans Blue solution (4 mL/kg) was administered via caudal vein injection. Six hours post-injection, transcardial perfusion with heparinized saline was performed. Cerebral tissues were homogenized in 50% trichloroacetic acid and centrifuged at 14,000 × g for 20 min (4°C). Absorbance of the supernatant was measured at 610 nm using a spectrophotometer. EB content was calculated (μg/g brain tissue) based on a standard curve, with triplicate experiments per group (n = 3).

**Terminal Deoxynucleotidyl Transferase dUTP Nick End Labeling (TUNEL) Staining:** Apoptotic cells were detected following manufacturer's protocol (Roche Diagnostics GmbH, Cat. No. 11684817910). TUNEL-positive nuclei were quantified using fluorescence microscopy.

**Hematoxylin and Eosin (H&E) Staining:** Tissue sections from heart, liver, spleen, lung, and kidney were processed with hematoxylin and eosin staining for histopathological evaluation.

**Enzyme-Linked Immunosorbent Assay (ELISA) for Inflammatory Cytokines:** *(In Vivo Study):* At 72 h post-surgery, mice (n = 3/group) were euthanized under isoflurane anesthesia. Blood samples were collected via retro-orbital puncture and allowed to clot for 2 h at room temperature. Serum was isolated by centrifugation at 3000 × g for 15 min. Serum concentrations of IL-10 and TNF-α were quantified using commercial ELISA kits (Gelatins Biotech Co., Ltd., Wuhan, China) following manufacturer's protocols. *(In Vitro Study):* PC12 cells subjected to oxygen-glucose deprivation/reperfusion (OGD/R) were treated with designated interventions. Cell culture supernatants were collected and centrifuged at 3000 × g for 15 min to remove cellular debris. IL-10 and TNF-α levels in supernatants were analyzed using identical ELISA methodology.

**Hemolysis Assay** *(Erythrocyte Suspension Preparation)*: Whole blood was collected from Sprague-Dawley rats via cardiac puncture. After centrifugation at 1500 × g for 10 min (4°C), plasma and the buffy coat were discarded. The erythrocyte pellet was triple-washed with 0.9% NaCl (1500 × g, 10 min/wash). A 2% erythrocyte suspension was prepared by mixing 200 μL packed red blood cells with 9.8 mL PBS (pH 7.4). *(Hemolysis Detection):* A 500 μL aliquot of erythrocyte suspension was combined with 500 μL test sample (diluted in 0.9% NaCl) and incubated at 37°C for 4 h. Following centrifugation (1500 × g, 10 min), supernatant absorbance was measured at 540 nm. Hemolysis percentage (%) was calculated as:

Hemolysis (%)=(Apositive control−Anegative control)/(Asample−Anegative control)×100, Hemolytic potential was defined as >5% hemolysis.

## 3 Results

### 3.1 Construction and mechanism of sivelestat-loaded neutrophil-targeted polymeric nanoparticles

A functional nanodrug delivery system was constructed via a three-step strategy (Fig. 1A). First, thioacetal (TK) served as an ROS-responsive linker to covalently conjugate poly(lactic-co-glycolic acid) (PLGA) with polyethylene glycol (PEG), forming an amphiphilic polymer (PLGA-TK-PEG-COOH) with a hydrophobic PLGA core and hydrophilic PEG shell. Subsequently, the formyl peptide receptor (FPR)-targeting peptide CFLFLF was directionally conjugated to the PEG terminus via carboxyl-amine coupling, endowing it with specific binding affinity to FPR on neutrophils. Finally, SIV was encapsulated into the hydrophobic PLGA core via nanoprecipitation to obtain targeted nanoparticles (T-SIV). Non-targeted nanoparticles (NT-SIV) were prepared by omitting the CFLFLF conjugation step, with all other synthesis parameters identical to T-SIV. Transmission electron microscopy (TEM) revealed that both T-SIV and NT-SIV exhibited monodisperse spherical structures with an average diameter of approximately 50-100 nm (Fig. 1B). The average hydrodynamic diameters of NT-SIV and T-SIV were determined using dynamic light scattering (DLS) (Fig. 1C), and the results show that the particle size of T-SIV is slightly larger than that of NT-SIV, which is consistent with the results obtained by TEM. The Zeta potential of NT-SIV and T-SIV were determined to be both negative, with zeta potential measurements indicated values of approximately −15 mV for NT-SIV and −17.5 mV for T-SIV indicating good colloidal stability (Supplementary Fig. 1). These results confirmed the successful preparation of T-SIV nanoparticles. The drug loading capacity, encapsulation rate of these nanoparticles, as well as the drug release kinetics under the action of ROS were simulated using Cy5 dye. The amount of SIV loading capacity of the nanoparticles is 85 mg/g, and the encapsulation efficiency of T-SIV was calculated to be 93.75% (Supplementary Fig. 2 and Supplementary Fig. 3). The drug release profile demonstrated a rapid release under the condition of the existence of ROS, however, when ROS is absent, the drug release enters a slow stage. After 72 h, the drug release reaching approximately 77%, revealing the ROS-responsive release characteristic of this delivery system (Supplementary Fig. 4).

### 3.2 Neutrophil-mediated targeted enrichment of T-SIV in cerebral ischemic regions

**Fig. 2:**
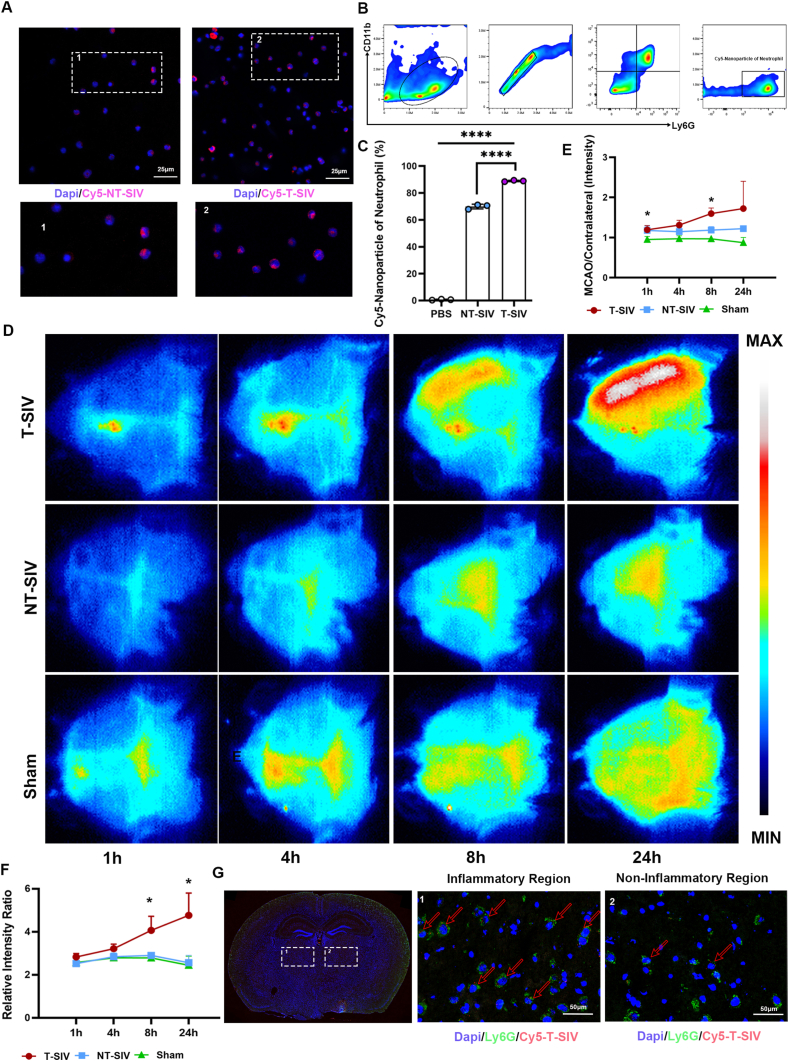
**Targeting validation of T-SIV nanoparticles.** (A) Comparative confocal imaging of Cy5-labeled T-SIV/NT-SIV uptake in murine bone marrow-derived neutrophils (red: Cy5; blue: DAPI). (B-C) Representative flow cytometry profiles of Cy5-labeled T-SIV nanoparticle uptake by murine bone marrow-derived neutrophils. Quantitative comparison of neutrophil uptake efficiency between T-SIV and NT-SIV groups (n = 3; ∗∗∗∗P < 0.0001 vs. PBS, One-way ANOVA + Tukey's post hoc test). (D) NIR-II in *vivo* imaging showing temporal-spatial distribution of T-SIV-IR780 (vs. NT-SIV-IR780 and Sham) in cerebral ischemic regions at 1, 4, 8, and 24 h post-injection (n = 3/group). (D-E) Quantitative analysis of ischemic-to-muscle (E) and ischemic-to-contralateral (F) fluorescence ratios (n = 3; ∗P < 0.05, T-SIV vs. NT-SIV and Sham, Kruskal-Wallis test). (G) Post-treatment immunofluorescence mapping of T-SIV distribution (red: Cy5-labeled T-SIV; green: Ly6G + neutrophils; blue: DAPI) in ischemic brain sections.

To validate the targeted enrichment capability of T-SIV in cerebral ischemic regions, we performed a series of experiments. In *vitro* co-incubation assays first assessed neutrophil uptake: Confocal images demonstrated Cy5-labeled T-SIV specifically bound to neutrophil membranes (Supplementary Fig. 5), with significantly higher fluorescence intensity than the NT-SIV group (Fig. 2A). Flow cytometry quantification confirmed an 88.9% uptake rate of T-SIV by neutrophils, substantially higher than NT-SIV and PBS groups (Fig. 2B-C), indicating enhanced cellular uptake via targeted modification. Subsequently, in *vivo* NIR-II imaging revealed significantly elevated fluorescence intensity in the ischemic region of T-SIV-treated MCAO mice at 4 h post-injection, peaking at 24 h (Fig. 2D–F). Quantitative analysis showed T-SIV fluorescence intensity at 8 h exceeded both NT-SIV and Sham groups (Fig. 2E). Confocal imaging of brain sections further confirmed greater T-SIV accumulation in ischemic regions versus non-ischemic areas and NT-SIV-treated ischemic regions (Fig. 2G, Supplementary Fig. 6). To investigate cellular-level targeting, a blood-brain barrier model was established using bEnd.3 cell monolayers (TEER ≥300 Ω cm^2^). Transwell migration assays demonstrated that neutrophils carrying Cy5-T-SIV in the upper chamber exhibited significantly higher CD11b + Cy5+ cell migration to the lower chamber – regardless of fMLP chemoattractant (100 nM) presence – compared to NT-SIV groups (Supplementary Fig. 7), consistent with in *vivo* distribution patterns. Furthermore, comprehensive safety assessments corroborated biocompatibility: H&E-stained organs showed no pathology (Supplementary Fig. 8); serum biomarkers (ALT, BUN, CK-MB) and hemolysis rates remained normal (Supplementary Figs. 9–10); while PC12 cell assays confirmed viability preservation (>85% survival at ≤50 μM) despite toxicity at 16 μM (Supplementary Fig. 11A–B), and significant cytoprotection under oxygen-glucose deprivation (OGD) by both free sivelestat (10 μM) and nanoformulations (Supplementary Fig. 11C–D). Collectively, these findings substantiate the targeted enrichment efficacy and biosafety profile of T-SIV.

### 3.3 T-SIV suppresses neutrophil infiltration, NETs formation, and neuroinflammation

**Fig. 3:**
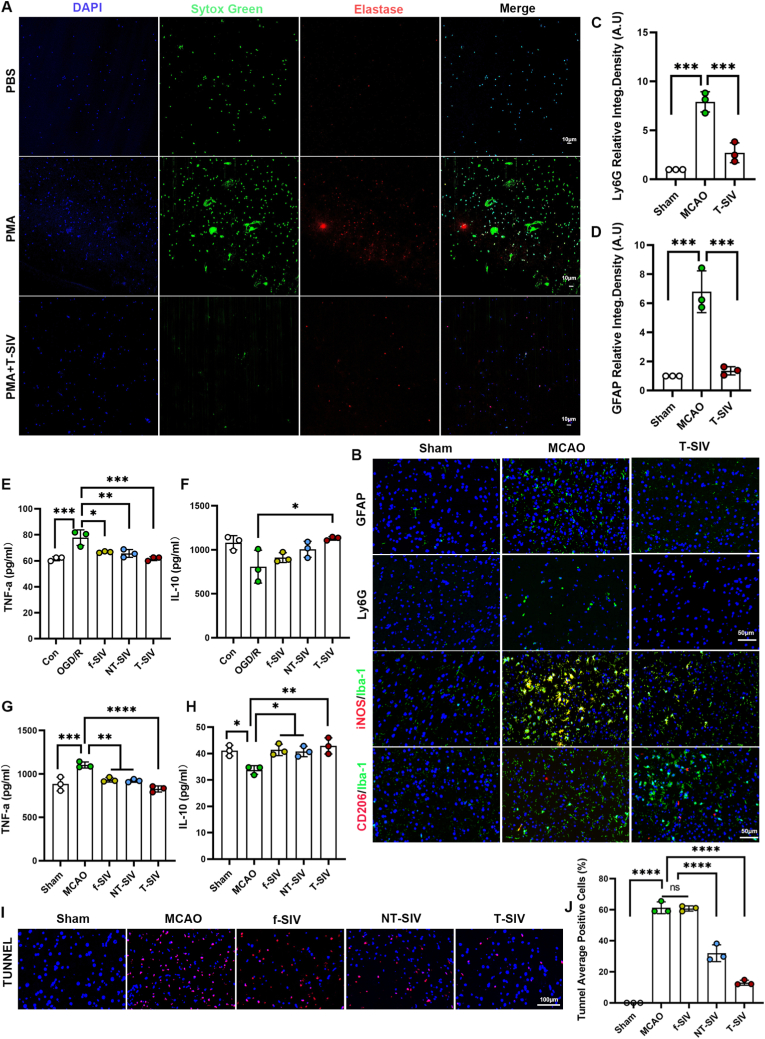
**T-SIV regulatory effects of Neutrophil Elastase (NE) Inhibition on NET Formation.** (A) Confocal immunofluorescence images showing colocalization of NET-associated DNA (SYTOX Green, green) and NE (red). (B) Immunofluorescence staining of brain sections (3 days post-modeling): GFAP (astrocytes, green), Ly6G (neutrophils, green), iNOS+/Iba1+ (M1 microglia, magenta), CD206+/Iba1+ (M2 microglia, red). (C-D) Quantitative fluorescence intensity analysis of Ly6G + neutrophils (C) and GFAP + astrocytes (D) (n = 3; ∗∗∗P < 0.001 vs. MCAO). (E-H) ELISA quantification of TNF-α/IL-10 levels in OGD/R-treated PC12 cell supernatants (n = 3; ∗P < 0.05, ∗∗P < 0.01, ∗∗∗P < 0.001, ∗∗∗∗P < 0.0001 vs. OGD/R). (G-H) Serum TNF-α/IL-10 levels in MCAO mice (n = 3; ∗P < 0.05, ∗∗P < 0.01, ∗∗∗P < 0.001, ∗∗∗∗P < 0.0001 vs. MCAO). (I) TUNEL staining of ischemic regions (red: TUNEL + nuclei; blue: DAPI). (J) Quantification of apoptotic cells (n = 3; ∗∗∗∗P < 0.0001 vs. MCAO). Statistical annotations: One-way ANOVA + Tukey's post hoc test.

In cerebral ischemic injury, neutrophils activated by chemokines release neutrophil extracellular traps (NETs), which exacerbate pathological progression through thrombosis promotion, inflammation aggravation, blood-brain barrier disruption, and neuronal apoptosis [28,29]. To evaluate the therapeutic value of inhibiting NETs formation, we isolated bone marrow-derived neutrophils from mice and stimulated them with PMA (100 nM) before T-SIV treatment. Notably, immunofluorescence revealed significantly weakened co-localization signals between SYTOX Green-labeled extracellular DNA and neutrophil elastase (NE) (Fig. 3A), while ELISA confirmed marked reduction in key NETs components (NE-DNA complexes) (Supplementary Fig. 12), and further ELISA analysis of MPO-DNA complexes, another specific marker of NETs, showed a significant decrease upon T-SIV treatment (Supplementary Fig. 14). collectively demonstrating T-SIV's potent NETs inhibition. Furthermore, in PC12 neuronal cells co-cultured with NETs, T-SIV treatment substantially enhanced cell viability (Supplementary Fig. 13). Extending to in *vivo* validation, brain sections showed significantly elevated GFAP^+^ astrocyte fluorescence intensity and Ly6G^+^ neutrophil infiltration density in MCAO versus Sham groups (Fig. 3B), whereas T-SIV treatment dramatically reduced both parameters (Fig. 3C–D), confirming its suppression of astrocyte activation and neutrophil infiltration. Concurrently, diminished iNOS^+^ (M1) and augmented CD206^+^ (M2) microglial signals in T-SIV-treated groups indicated promoted phenotypic switching from pro-inflammatory to anti-inflammatory states (Fig. 3B). Critically, TUNEL staining revealed significantly fewer apoptotic cells in ischemic regions of T-SIV-treated mice versus MCAO controls, outperforming both f-SIV and NT-SIV groups (Fig. 3I–J). Mechanistically, ELISA demonstrated markedly decreased TNF-α and elevated IL-10 levels in serum/cell supernatants post-T-SIV treatment (Fig. 3E–H), ultimately establishing its bidirectional modulation of inflammatory cytokines in mitigating neuroinflammation across experimental models.

### 3.4 T-SIV attenuates cerebral ischemia-reperfusion injury and promotes functional recovery

**Fig. 4:**
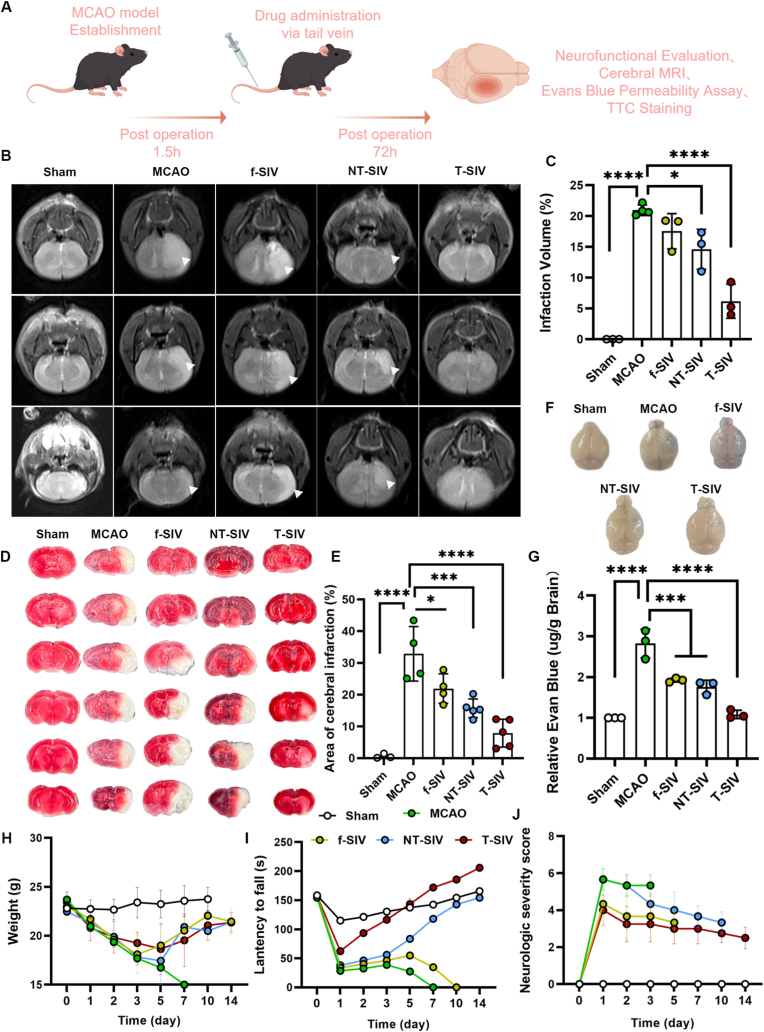
**Multidimensional Protective Effects of T-SIV against Cerebral Ischemia-Reperfusion Injury.** (A) Schematic workflow of experimental design. (B) Representative T2-weighted MRI scans at postoperative day 3 (red arrow: hyperintense edema regions). (C) Volumetric analysis of MRI T2 hyperintensity (n = 3; ∗∗P < 0.01, ∗∗∗∗P < 0.0001 vs. MCAO). (D) TTC-stained brain sections demonstrating infarct recovery (white: infarcted tissue; red: viable parenchyma). (E) Quantification of TTC-defined infarct areas (n = 3; ∗∗∗P < 0.001, ∗∗∗∗P < 0.0001 vs. MCAO). (F) Evans Blue (EB) extravasation mapping blood-brain barrier (BBB) integrity (blue intensity correlates with permeability). (G) Quantitative EB content in ischemic hemispheres (n = 3; ∗∗∗P < 0.001, ∗∗∗∗P < 0.0001 vs. MCAO). (H) Postoperative body weight trajectories over 14 days. (I-J) Neurological recovery assessed by rotarod performance (I) and modified neurological severity scores (mNSS, J) (n = 3; ∗P < 0.05, ∗∗P < 0.01, ∗∗∗P < 0.001 vs. MCAO). Statistical Methods: Panels C-G: One-way ANOVA with Tukey's post hoc test, Panels H-J: Two-way ANOVA with Tukey's post hoc test.)

Following 72-h treatment in MCAO/R models (Fig. 4A) – where mice subjected to 1.5 h middle cerebral artery occlusion received intravenous injections (3 mg/kg) of respective formulations (Sham, MCAO, f-SIV, NT-SIV, T-SIV) – comprehensive assessments revealed multi-level neuroprotection. Initially, cerebral MRI T2-weighted imaging demonstrated significantly reduced edema volume in T-SIV-treated mice versus comparator groups (Fig. 4B–C). Concurrently, TTC staining quantification showed diminished infarction areas across treatment groups relative to MCAO controls, with T-SIV achieving maximal tissue preservation (Fig. 4D–E). Complementing these structural improvements, Evans Blue assays confirmed substantially restored blood-brain barrier integrity in T-SIV group (Fig. 4F–G). Critically, behavioral metrics demonstrated superior functional recovery: T-SIV-treated mice regained near-baseline body weight (Fig. 4H), exhibited prolonged rotarod endurance (Fig. 4I), and achieved the lowest modified Neurological Severity Scores (mNSS) among intervention groups (Fig. 4J). Collectively, these integrated findings establish T-SIV's efficacy in mitigating cerebral ischemia-reperfusion injury through enhanced neuroprotective mechanisms.

### 3.5 Molecular mechanisms underlying T-SIV-mediated suppression of neuroinflammation and apoptosis

**Fig. 5:**
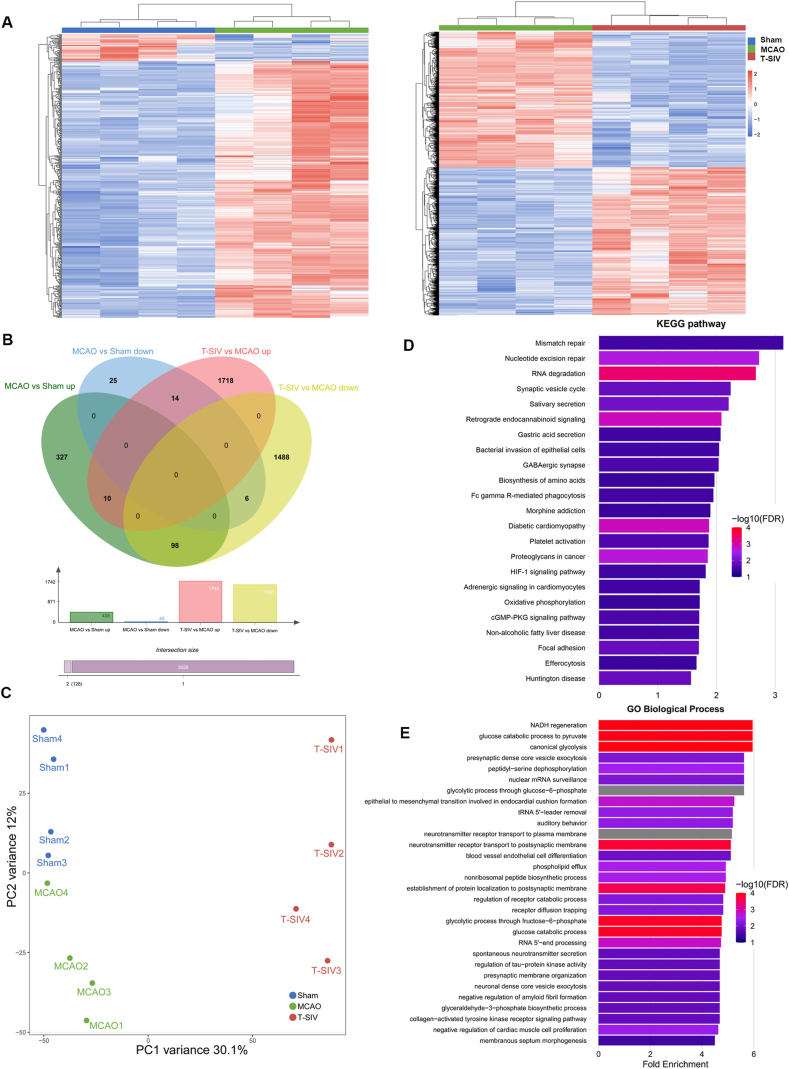
**Identification of Differentially Expressed Genes (DEGs).** (A) Hierarchical clustering of DEGs across Sham, MCAO, and T-SIV groups (two biological replicates shown). (B) Venn diagram illustrating overlapping DEGs between MCAO vs. Sham, and T-SIV vs. MCAO. (C) Principal component analysis (PCA) depicting sample clustering among experimental groups. (D-E) KEGG pathway (D) and GO biological process (E) enrichment analyses specifically targeting DU-pattern (downregulated-upregulated) and UD-pattern (upregulated-downregulated) genes identified in DEG intersection studies.

To elucidate T-SIV's therapeutic mechanisms against cerebral ischemia-reperfusion injury, we conducted RNA sequencing on brain tissues from Sham, MCAO, and T-SIV-treated groups. Applying differential expression gene (DEG) screening criteria (|fold change| > 2.0, adjusted ∗p < 0.05; Fig. 5A), we identified 480 DEGs in MCAO versus Sham (435 upregulated, 45 downregulated), while T-SIV versus MCAO comparison revealed 3334 DEGs (1742 upregulated, 1592 downregulated; Fig. 5B). Notably, principal component analysis (PCA) positioned T-SIV samples between Sham and MCAO clusters (PC1: 30.1%, PC2: 12%; Fig. 5C), indicating partial reversal of ischemia-induced transcriptional alterations. Through DEG intersection analysis, 98 genes exhibited upregulated-downregulated (UD) patterns (further upregulated by T-SIV after MCAO induction), whereas 14 genes displayed downregulated-upregulated (DU) patterns (further downregulated post-T-SIV). Subsequent KEGG pathway analysis demonstrated robust enrichment in: synaptic vesicle cycling, GABAergic synapse signaling, cGMP-PKG signaling pathway, and FcγR-mediated phagocytosis (Fig. 5D). Complementarily, GO biological process analysis revealed significant involvement in glucose catabolism to pyruvate, presynaptic dense-core vesicle exocytosis, and negative regulation of amyloid fibril formation (Fig. 5E). Collectively, these multi-omics findings suggest T-SIV mitigates ischemic damage by synergistically modulating energy metabolism, synaptic function, and inflammatory pathways.

## 4 Discussion

Ischemic stroke, as a leading cause of disability in working-age populations, has become a focal area in global public health research. This study demonstrates that intravenously administered sivelestat-loaded polymeric nanoparticles (T-SIV) effectively cross the blood-brain barrier (BBB) through neutrophil-mediated targeting, achieving ischemic lesion-specific accumulation. Subsequently, T-SIV attenuates cerebral ischemia-reperfusion injury by suppressing neutrophil extracellular trap (NET) formation, mitigating neuroinflammation, and enhancing anti-inflammatory responses, ultimately promoting neurological recovery.

Neutrophils serve as critical biological vectors in this therapeutic strategy. We innovatively exploit their intrinsic recruitment to ischemic regions through a “hitchhiking” delivery approach. This system overcomes the inherent BBB penetration limitations of conventional therapeutics while demonstrating superior tissue targeting. Formyl peptide receptors (FPRs), neutrophil-specific surface targets, mediate cellular activation, migration, and NETosis during inflammatory cascades. These findings align with the mechanisms of polylactic-co-glycolic acid nanoparticles regulating neutrophil CNS infiltration reported by Satio et al. [30]. Notably, recent advances in targeting NETosis for stroke therapy have emerged: He et al. [12]systematically elucidated the triple detrimental mechanisms of NETs inischemic stroke (promoting lysis-resistant thrombi, disrupting BBB integrity, and amplifying neuroinflammation); Yin et al. [13] developed a neutrophil-hijacking nanoplatform that reprograms NETosis for targeted microglia polarization; Tang et al. [14]constructed an elastase-targeting biomimetic nanoplatform that inhibits NETosis and AIM2 inflammasome activation, promoting neurovascular remodeling. Furthermore, Mu et al.^31^ [][][]reported neutrophil-targeting platforms and pH-triggered polymersomes, respectively, for NET clearance and brain injury amelioration. Our T-SIV strategy echoes these cutting-edge developments but distinguishes itself by leveraging the ‘hitchhiking’ mechanism of neutrophils as natural carriers for ROS-responsive drug release. Experimental data further reveal that T-SIV significantly enhances neutrophil binding efficiency via FPR-mediated targeting, thereby inhibiting NET-associated signaling pathways, reducing vascular basement membrane degradation, and ultimately restricting parenchymal infiltration of inflammatory cells including astrocytes and microglia. Moreover, T-SIV treatment markedly decreases pro-inflammatory cytokine levels in ischemic regions while promoting microglial polarization toward anti-inflammatory phenotypes, corroborating findings from Ichiro Horinokita's team [32]. Multimodal assessments (neurological scoring, survival analysis, Evans Blue permeability, TTC staining, and MRI) collectively demonstrate T-SIV's superior efficacy in alleviating post-ischemic neurological deficits, potentially mediated through NETs clearance. In addition, we performed a 14-day long-term observation: T-SIV treated mice maintained significantly lower mNSS scores, recovered body weight close to baseline, and exhibited prolonged rotarod latency throughout the 14-day period, confirming sustained neuroprotection for at least 14 days. These results reaffirm NETs' pivotal role in ischemia-reperfusion pathology, consistent with our previous research [31]. Crucially, free sivelestat (f-SIV) failed to show therapeutic benefits due to poor BBB penetration and lack of cellular targeting, mirroring Okeke et al.’s observations [19].

Several limitations of the present study should be acknowledged. First, the colloidal stability of T-SIV in physiological media and a strict negative control for the ROS-triggered release assay were not included, although indirect evidence supports ROS responsiveness and stability. Second, the in vitro BBB model using bEnd.3 monolayers has lower tightness than the intact BBB, and an NT-SIV control was missing in Fig. 3A; these aspects limit the direct comparison but do not affect the main conclusions. Third, microglial polarization was assessed only by qualitative staining of iNOS and CD206 without quantitative analysis or additional markers, so the M1-to-M2 shift should be considered a trend. These limitations do not invalidate the main conclusions but highlight areas for future improvement. Despite certain limitations—specifically, the need to further elucidate the molecular mechanisms of Sivelestat-mediated NET clearance in neuroprotection and to investigate the pathological roles of other immune cells (e.g., macrophages) during cerebral ischemia-reperfusion injury—this study demonstrates that an FPR-targeted nanodrug delivery system significantly enhances the therapeutic efficacy of sivelestat. To our knowledge, this is one of the few studies to leverage neutrophil hitchhiking for sivelestat delivery in the treatment of ischemic stroke. Future investigations will prioritize: (1) delineating precise signaling pathways governing T-SIV-modulated NETs dynamics; (2) evaluating clinical translatability in higher-order mammalian models; and (3) exploring synergistic therapeutic interactions with complementary anti-inflammatory modalities.

## 5 Conclusion

This study demonstrates that the polymer-based T-SIV nanoparticles significantly enhance therapeutic efficacy against cerebral ischemia-reperfusion injury through dual mechanisms: inhibition of NE activity to suppress pathological NETs formation, and attenuation of neuroinflammation via phenotypic reprogramming of immune cells from pro-inflammatory to anti-inflammatory states. These findings not only expand the mechanistic understanding of Sivelestat's therapeutic potential in ischemic stroke but also establish a novel drug delivery paradigm for clinical translation. Future investigations should prioritize: (1) molecular-level elucidation of NE-NETs axis regulation; (2) systematic evaluation of long-term biosafety profiles; and (3) nanocarrier engineering optimization to expedite clinical development.

## CRediT authorship contribution statement

**Shuyu Wu:** Data curation, Investigation, Methodology, Resources, Visualization, Writing – original draft. **Xiaoli Sun:** Formal analysis, Visualization. **Xiai Luo:** Data curation, Formal analysis, Methodology. **Yang Li:** Data curation, Formal analysis, Methodology. **Jinlong Wan:** Data curation, Formal analysis. **Chuanwu Xiong:** Data curation, Formal analysis. **Qingmin Chen:** Methodology, Software. **Xiaohong Lv:** Resources, Software. **Ruwei Jie:** Investigation, Methodology. **Sainawar Tursun:** Investigation, Methodology. **Jianjun Ma:** Funding acquisition, Supervision, Validation. **Qingchun Mu:** Funding acquisition, Resources, Supervision, Visualization. **Longguang Tang:** Funding acquisition, Project administration, Resources, Supervision. **Hongzhi Gao:** Supervision, Writing – review & editing.

## Declaration of competing interest

The authors declare that they have no known competing financial interests or personal relationships that could have appeared to influence the work reported in this paper.

## Data Availability

Data will be made available on request.
