# Supplementary material for: Neutrophil-targeted nanoparticles delivering sivelestat alleviate cerebral ischemia-reperfusion injury by suppressing NETosis
Source: Mater Today Bio. 2026 Jun 29;39:103408. doi: 10.1016/j.mtbio.2026.103408 (PMC13334822; doi:10.1016/j.mtbio.2026.103408)
Supplement: Multimedia component 1 [file mmc1.docx]

**Neutrophil-targeted nanoparticles delivering** **sivelestat alleviate cerebral ischemia-reperfusion injury by suppressing NETosis**

Shuyu Wu^1,4,#^, Xiaoli Sun^2,3,#^, Xiai Luo^2,#^, Yang Li^3,#^, Jinlong Wan^5^, Chuanwu Xiong^2^, Qingmin Chen^8^, Xiaohong Lv^8^, Ruwei Jie^5^, Sainawar Tursun^5^, Jianjun Ma^6,7,^*, Qingchun Mu^5,^*, Longguang Tang^3,^*, Hongzhi Gao^1,^*

^1^Department of Neurosurgery, The Second Affiliated Hospital of Fujian Medical University, Quanzhou, China, 362000

^2^Hunan Province Key Laboratory for Synthetic Biology of Traditional Chinese Medicine, School of Pharmaceutical Sciences, Hunan University of Medicine, Huaihua, 418000, China

^3^Department of Pharmacy, Center for Regenerative and Aging Medicine, the Fourth Affiliated Hospital of School of Medicine, and International School of Medicine, International Institutes of Medicine, Zhejiang University, Yiwu, China, 322000

^4^Department of Neurosurgery, Hainan General Hospital, Haikou, Hainan, China, 570000

^5^Affiliated Gaozhou People’s Hospital, Guangdong Medical University, Maoming 525200, China

^6^Department of Orthopaedic Surgery, Sir Run Shaw Hospital, Zhejiang University School of Medicine, Zhejiang Key Laboratory of Mechanism Research and Precision Repair of Orthopaedic Trauma and Aging Diseases, Hangzhou, Zhejiang, China, 310000

^7^Department of Orthopaedic Surgery, the Fourth Affiliated Hospital of School of Medicine, and International School of Medicine, International Institutes of Medicine, Zhejiang University, Yiwu, China, 322000

^8^Shanxi Province Cancer Hospital, Shanxi Hospital Affiliated to Cancer Hospital, Chinese Academy of Medical Sciences, Cancer Hospital Affiliated to Shanxi Medical University, Taiyuan, China, 030000

^#^These authors contributed equally.

*Corresponding authors. Email: [sealteam@zju.edu.cn](mailto:sealteam@zju.edu.cn) (J.M.); [muq@suda.edu.cn](mailto:muq@suda.edu.cn) (Q.M.); [tanglongguang@zju.edu.cn](mailto:tanglongguang@zju.edu.cn) (L.T.); [gaohongzhi@fjmu.edu.cn](mailto:gaohongzhi@fjmu.edu.cn) (H.G.);


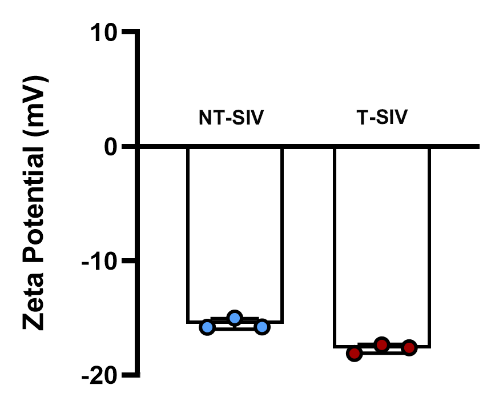


**Supplementary Figure 1**. Negative surface charges of both formulations confirmed by zeta potential analysis.


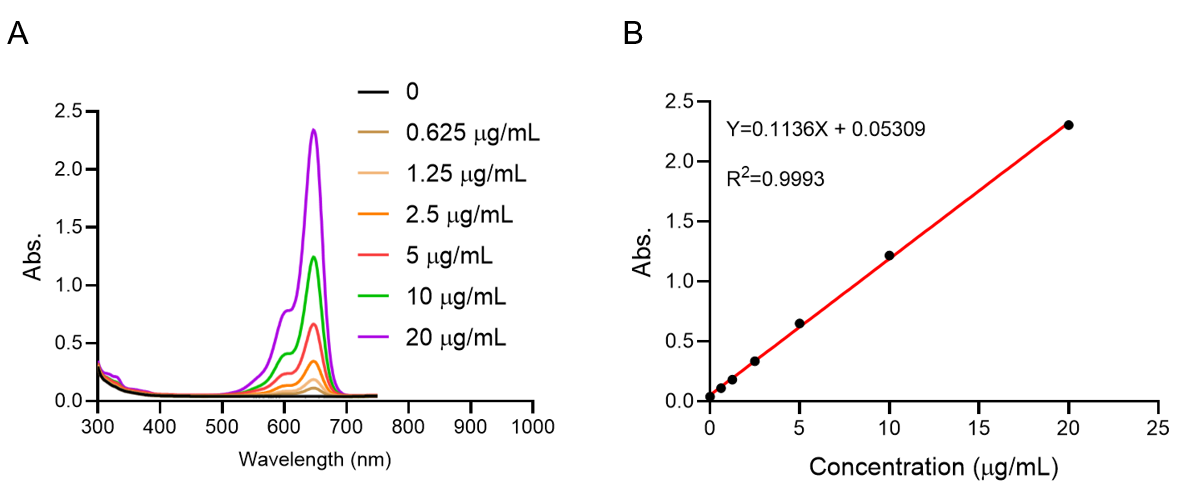


**Supplementary Figure 2**. (A) UV-visible absorption spectra of Cy5 on different concentration. (B)The standard curves of UV-via absorption values of Cy5.


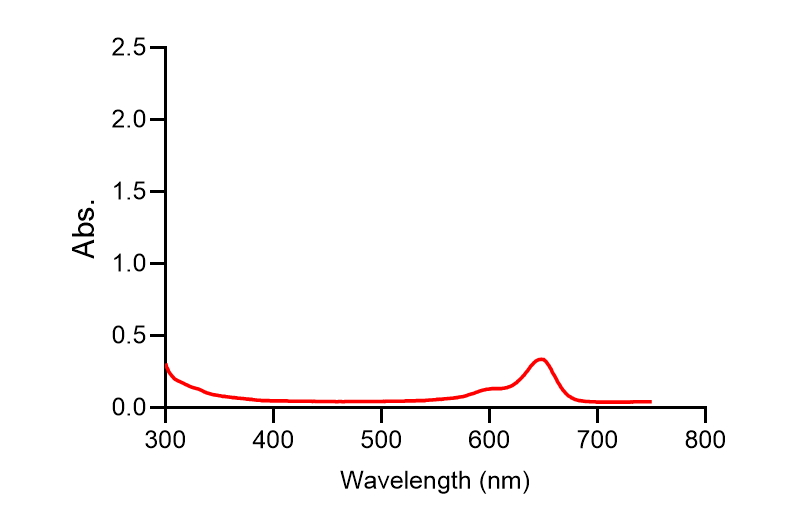


**Supplementary Figure 3**. UV-visible absorption spectra of Cy5 in the supernatant solution after loading.

The equation of Cy5’s standard curve is Y = 0.1136X + 0.05309. The R^2^ = 0.9993, where X is the concentration of Cy5, and Y is the absorbance. drug loading capacity encapsulation yield

The equation drug loading capacity is: $=$ $\frac{\left( 1000-\frac{Y-0.05309}{0.1136}*25 \right)}{11000}*100\%$

The equation drug encapsulation yield is: $=$ $\frac{\left( 1000-\frac{Y-0.05309}{0.1136}*25 \right)}{1000}*100\%$

where the mass of total Cy5 is 1 mg, the mass of total Cy5-PLGA-TK-pep nanoparticles is 10 mg, the volume of total Cy5-PLGA-TK-pep nanoparticles is 25 mL and Y is the absorbance of the Cy5 in the supernatant.


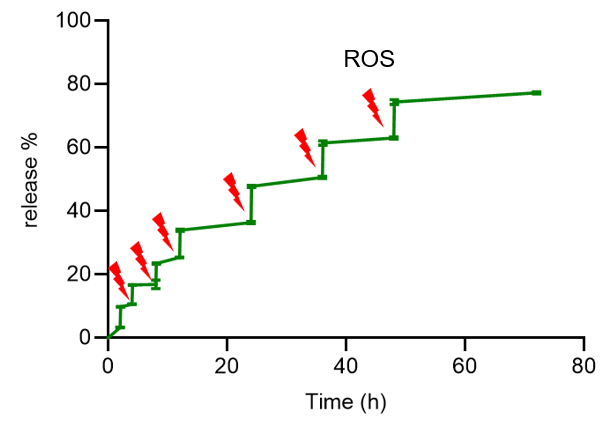


**Supplementary Figure 4**. SIV release from T-SIV NPs under the condition of the existence of ROS (H_2_O_2_) in 72 h.


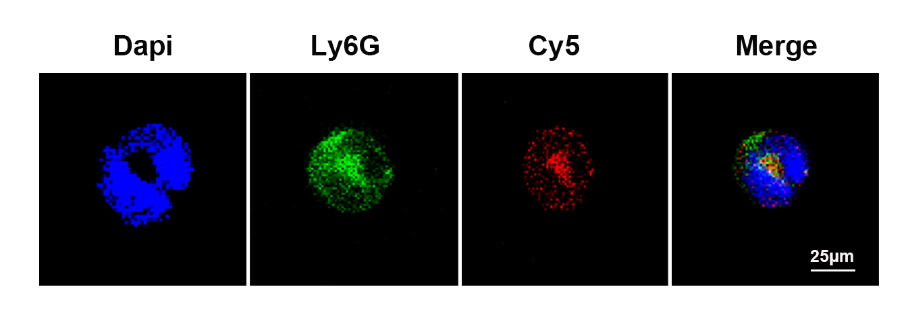


**Supplementary Figure 5**. Confocal microscopy imaging of T-SIV-neutrophil interactions (red: Cy5-labeled T-SIV; blue: DAPI-stained nuclei).


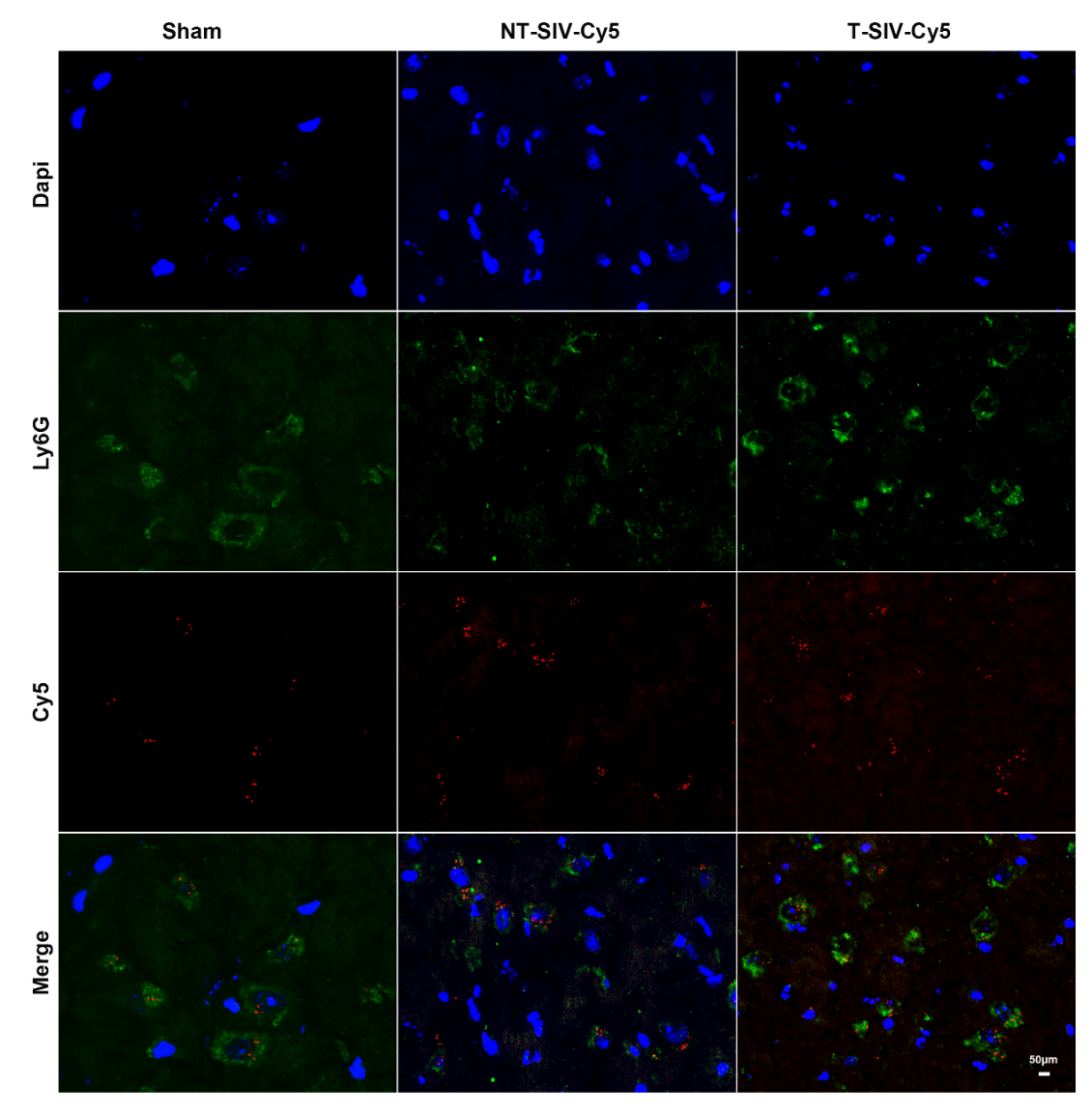


**Supplementary Figure 6. In *vivo* immunofluorescence confocal imaging demonstrating neutrophil-nanoparticle co-localization in cerebral sections across experimental groups.** (red: Cy5-labeled T-SIV/NT-SIV; green: Ly6G+ neutrophils; blue: DAPI-stained nuclei).


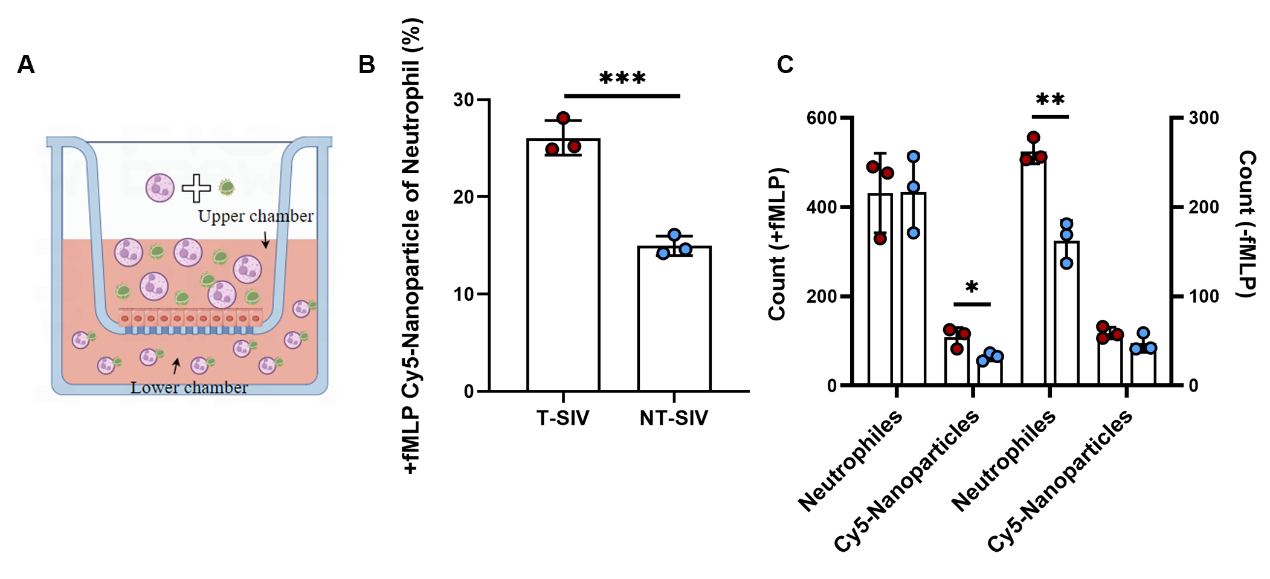


**Supplementary Figure 7. (A) Schematic of Transwell migration assay design. (B) Flow cytometric quantification of neutrophil transmigration efficiency (CD11b+Cy5+ cells) under fMLP (100 nM) stimulation. T-SIV group exhibited significantly enhanced penetration compared to NT-SIV controls (n = 3; ***P < 0.001 by one-way ANOVA with Tukey’s post hoc test). (C) Comparative neutrophil transmigration across bEnd.3 monolayers with/without fMLP chemoattraction (n = 3; **P < 0.01, *P < 0.05 vs. NT-SIV). Statistical analysis: One-way analysis of variance (ANOVA) with Tukey’s post hoc test.**


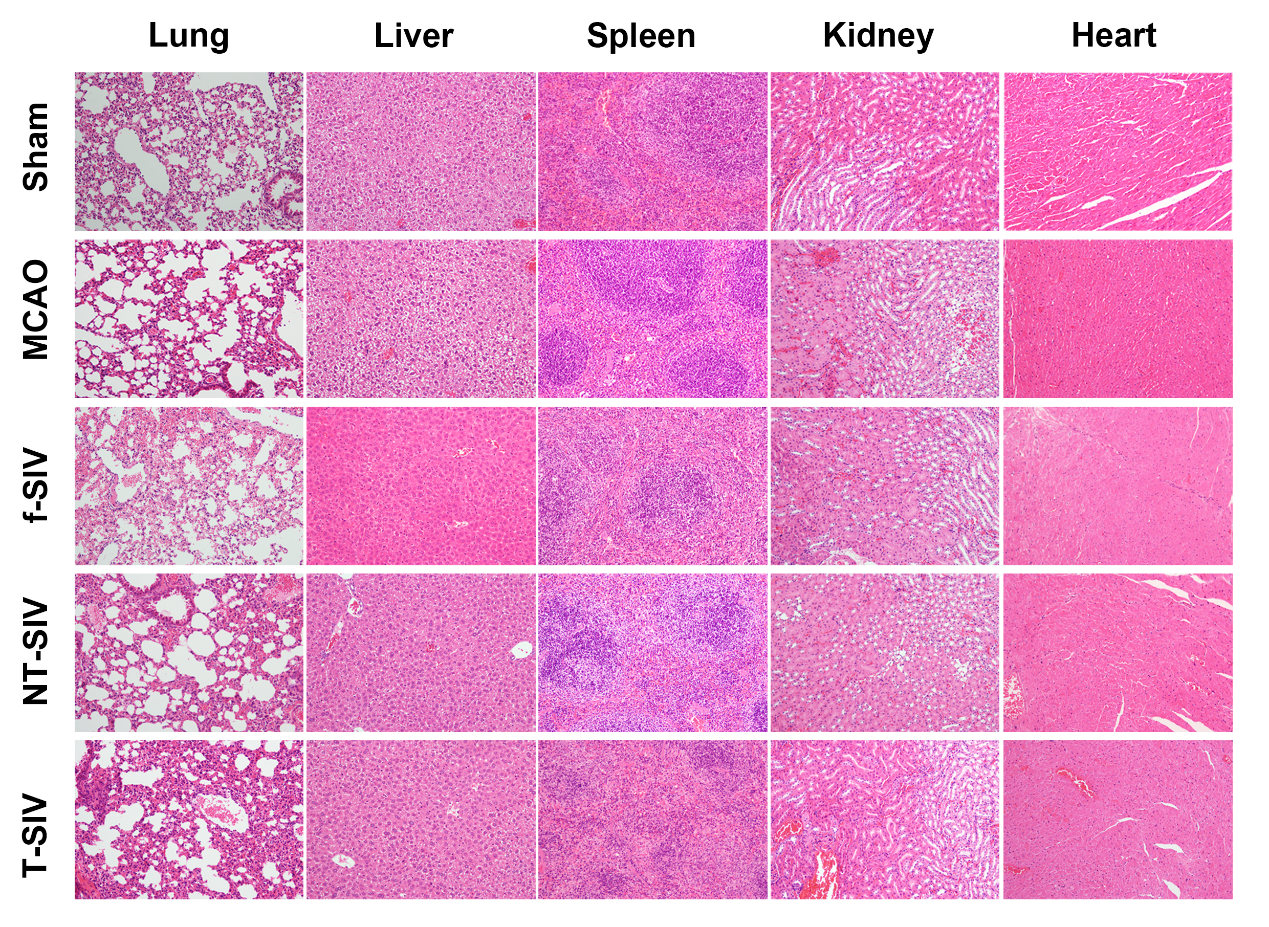


**Supplementary Figure 8. Histopathological assessment of major organ sections across experimental groups by H&E staining.** Representative micrographs of pulmonary, hepatic, splenic, renal, and myocardial tissue sections.


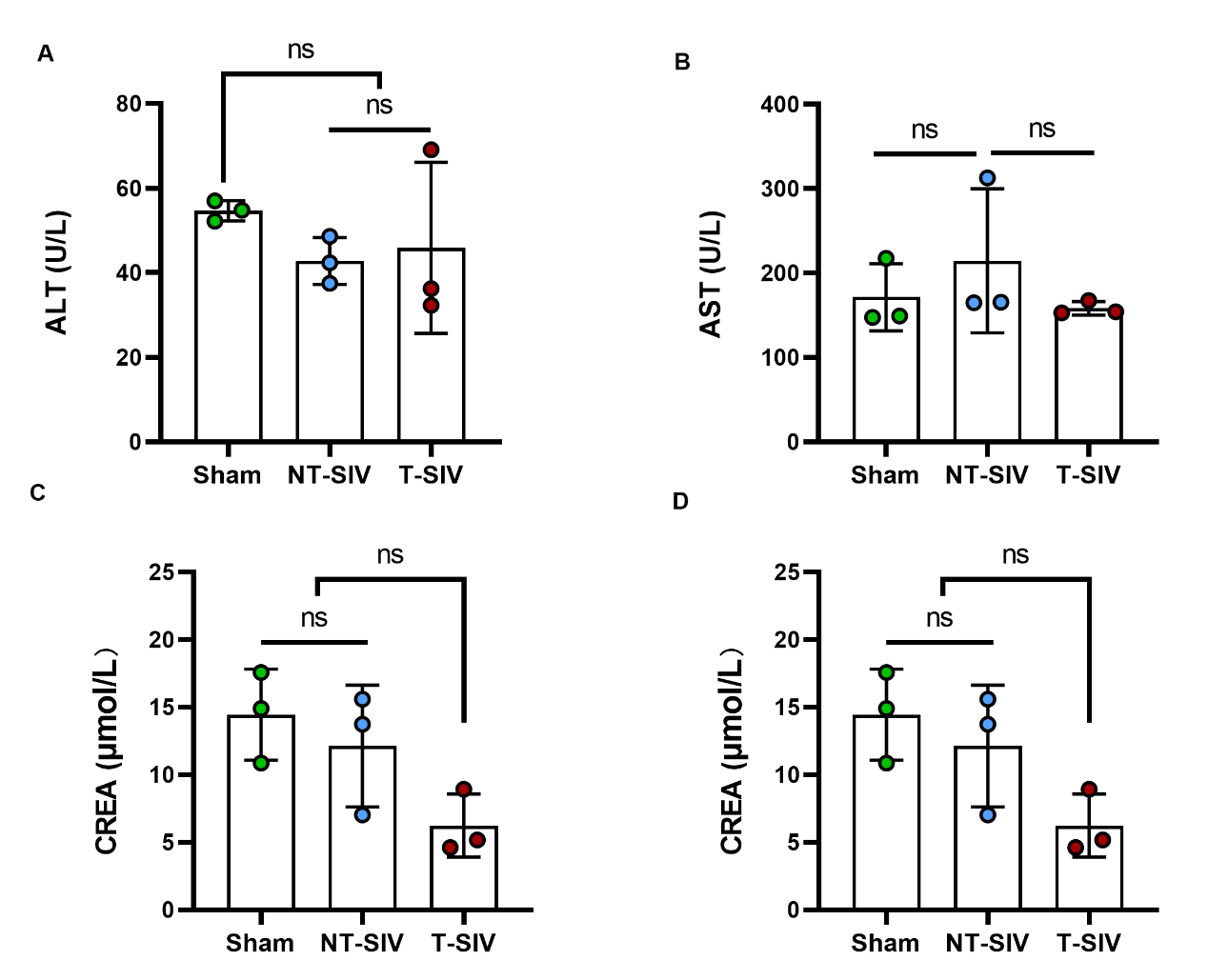


**Supplementary Figure 9. Serum biochemical profiling of Sham, NT-SIV, and T-SIV groups.** Quantification of hepatic function markers (ALT, AST), renal parameters (CREA), and myocardial injury biomarker (CK-MB) demonstrates no significant intergroup variations (n = 3; P > 0.05). Statistical analysis: One-way analysis of variance (ANOVA) with Tukey’s post hoc test.


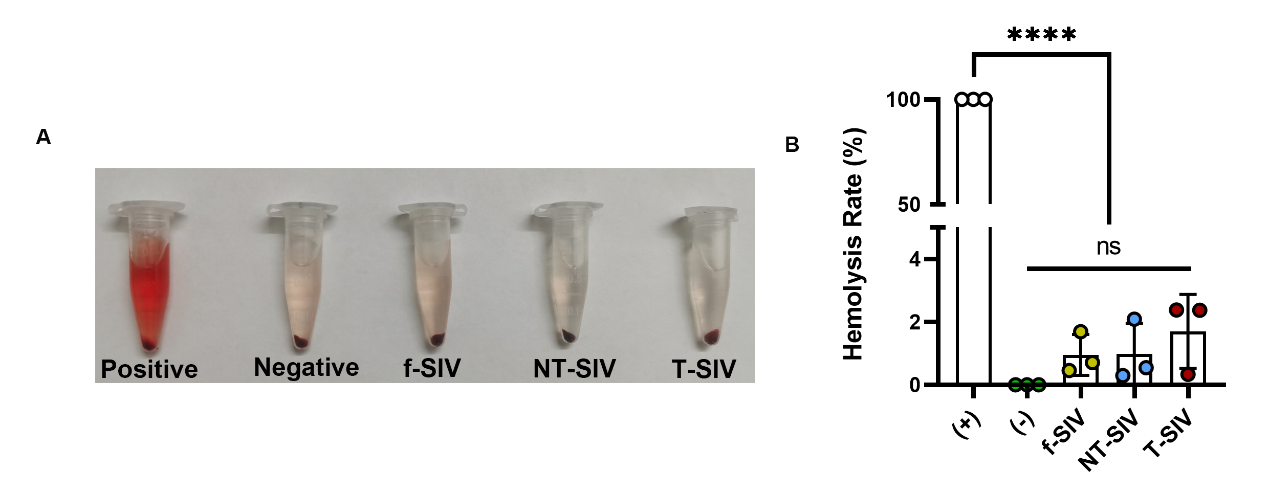


**Supplementary Figure 10. Hemocompatibility assessment via erythrocyte lysis assay.** All tested formulations (T-SIV, NT-SIV, f-SIV) exhibited hemolysis rates <5% (n = 3; PBS: negative control [NC]; ddH₂O: positive control [PC]), confirming excellent blood compatibility.


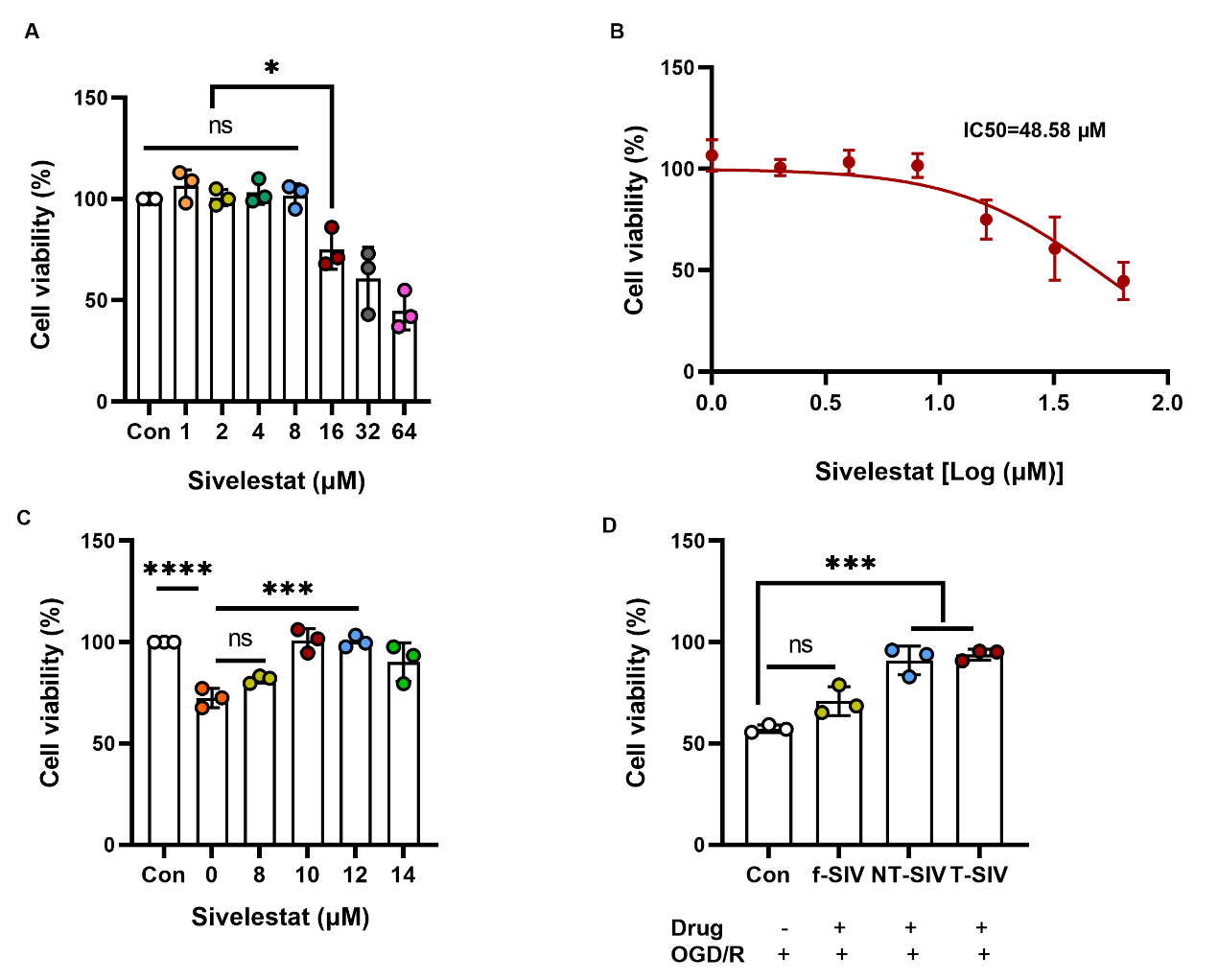


**Supplementary Figure 11. Cytotoxicity Assessment Using CCK-8 Assay. (A)** Viability of SIV-treated PC12 cells. **(B)** Determination of SIV's half-maximal inhibitory concentration (IC₅₀) against PC12 cells. **(C)** Optimal therapeutic concentration of SIV for PC12 cells based on viability profiles. **(D)** Cytoprotective effects of SIV formulations on PC12 cells subjected to oxygen-glucose deprivation/reperfusion (OGD/R) injury (n = 3; *P < 0.05, **P < 0.01, ***P < 0.001, ****P < 0.0001 vs. control group). Statistical significance was determined by one-way analysis of variance (ANOVA) with Tukey’s multiple comparison test.


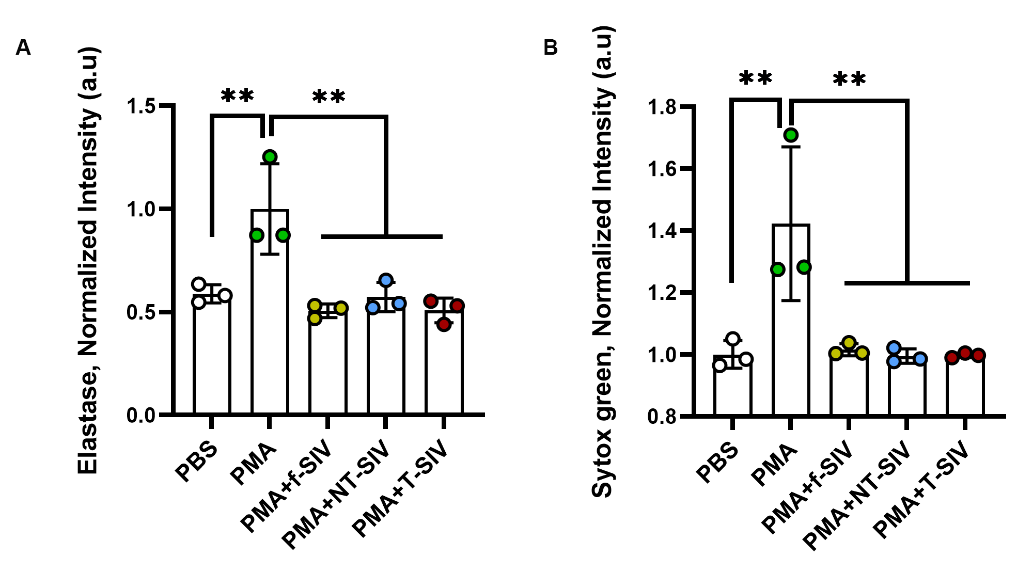


**Supplementary Figure 12.** Quantitative analysis of NET formation in PMA (100 nM)-stimulated murine neutrophils treated with/without SIV (10 μM) for 4 h: extracellular DNA levels measured via Sytox Green (1 μM) and NE activity assessed using the fluorogenic substrate Z-Ala-Ala-Ala-2Rhodamine110 (0.5 mM) (n = 3; ****P < 0.0001 vs. PMA group, microplate reader detection).


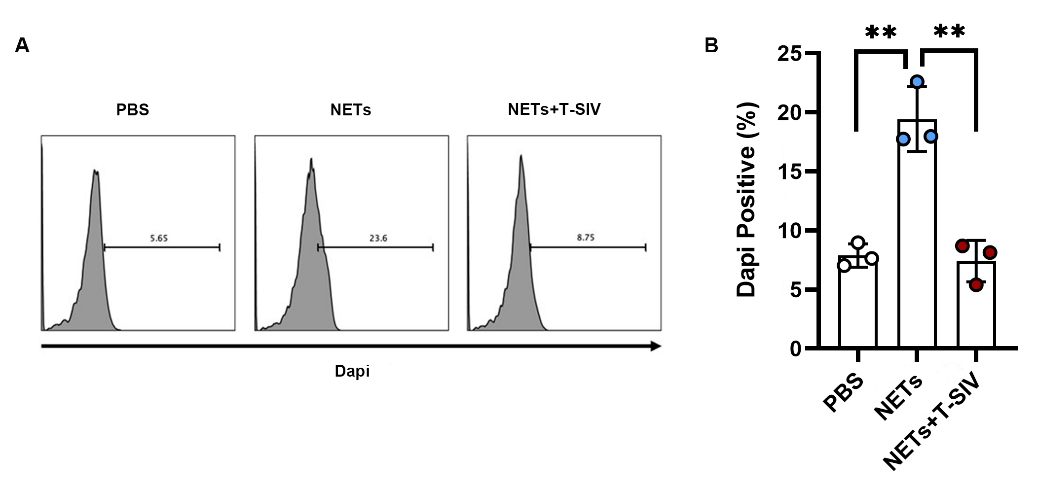


**Supplementary Figure 13.** Viability of PC12 cells treated with neutrophil NET supernatants (containing 5 μg/mL DNA) for 12 h in the presence/absence of SIV (representative data from triplicate experiments, n = 3; ****P < 0.0001 vs. NETs group). Statistical analysis: One-way analysis of variance (ANOVA) with Tukey’s multiple comparison test.


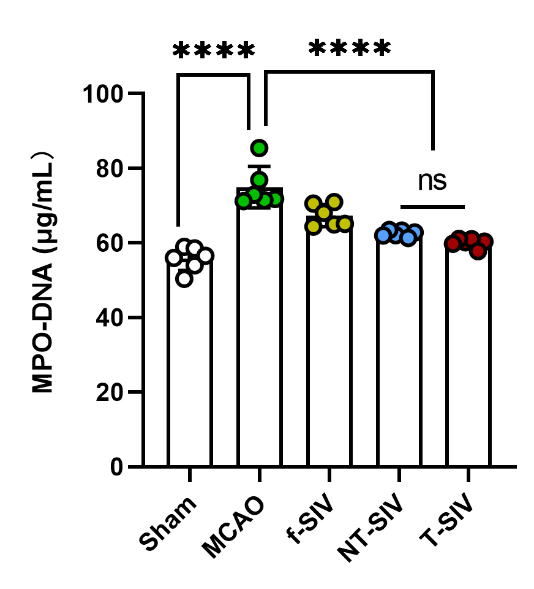


**Supplementary Figure 14. MPO-DNA levels in the ipsilateral brain tissue of C57 mice at 24 h after MCAO and treatment. Ipsilateral brain tissues were harvested 24 h post‑surgery, and MPO-DNA levels were quantified by ELISA (n = 6; ****P < 0.0001 vs. MCAO group).** Statistical analysis: One-way analysis of variance (ANOVA) with Tukey’s multiple comparison test.
